# Packaging Covered with Antiviral and Antibacterial Coatings Based on ZnO Nanoparticles Supplemented with Geraniol and Carvacrol

**DOI:** 10.3390/ijms22041717

**Published:** 2021-02-09

**Authors:** Małgorzata Mizielińska, Paweł Nawrotek, Xymena Stachurska, Magdalena Ordon, Artur Bartkowiak

**Affiliations:** 1 Center of Bioimmobilisation and Innovative Packaging Materials, Faculty of Food Sciences and Fisheries, West Pomeranian University of Technology Szczecin, Janickiego 35, 71-270 Szczecin, Poland; magdalena.ordon@zut.edu.pl (M.O.); artur.bartkowiak@zut.edu.pl (A.B.); 2 Department of Microbiology and Biotechnology, Faculty of Biotechnology and Animal Husbandry, West Pomeranian University of Technology, Szczecin, Nanotechnology Center for Education and Research, Piastów Avenue 45, 70-311 Szczecin, Poland; pawel.nawrotek@zut.edu.pl (P.N.); xymena.stachurska@zut.edu.pl (X.S.)

**Keywords:** SARS-Co-V2, phi 6 phage, antiviral properties, antibacterial properties, active coatings, active packaging

## Abstract

The purpose of the study was to obtain an external coating based on nanoparticles of ZnO, carvacrol, and geraniol that could be active against viruses such as SARS-Co-V2. Additionally, the synergistic effect of the chosen substances in coatings was analyzed. The goal of the study was to measure the possible antibacterial activity of the coatings obtained. Testing antiviral activity with human pathogen viruses, such as SARS-Co-V2, requires immense safety measures. Bacteriophages such as phi 6 phage represent good surrogates for the study of airborne viruses. The results of the study indicated that the ZC1 and ZG1 coatings containing an increased amount of geraniol or carvacrol and a very small amount of nanoZnO were found to be active against Gram-positive and Gram-negative bacteria. It is also important that a synergistic effect between these active substances was noted. This explains why polyethylene (PE) films covered with the ZC1 or ZG1 coatings (as internal coatings) were found to be the best packaging materials to extend the quality and freshness of food products. The same coatings may be used as the external coatings with antiviral properties. The ZC1 and ZG1 coatings showed moderate activity against the phi 6 phage that has been selected as a surrogate for viruses such as coronaviruses. It can be assumed that coatings ZG1 and ZC1 will also be active against SARS-CoV-2 that is transmitted via respiratory droplets.

## 1. Introduction

The packaging material containing antibacterial substances incorporated into the polymer matrix has begun to receive more attention for its use as anti-bacterial control agents in food packaging systems. It can ensure microbial food safety and extend the product shelf life through the inhibition of microorganism growth that is present on the surface of food products [1,2,3]. Many studies have demonstrated that zinc oxide nanoparticles are active against Gram-positive and Gram-negative bacteria, as well as against spores that are resistant to high temperature or high pressure [2,3,4,5], as well as yeasts and molds [2,6]. Zinc Oxide nanoparticles have been added to synthetic polymers using conventional incorporation methods, such as melt mixing or solvent casting, together with being added to compostable polymers, e.g., PLA, PHA [7]. Nanoparticles were also introduced into polymer coating layers (hydrophilic and hydrophobic) of antibacterial or antifungal packaging [2,3,8]. Several studies have shown an increase in the shelf life of food products packed in films containing ZnO nanoparticles (within a polymer matrix), or used with coatings containing ZnO nanoparticles [9,10].

An excellent example is an earlier study carried out by the author, which determined that boxes containing polyethylene films covered with a coating consisting of methyl-hydroxy-propyl-cellulose (MHPC) with ZnO nanoparticles were found to be the most effective packaging material to extend the quality and freshness of cod fillets after 144 h of storage at 5 °C [11].

Carvacrol is a natural, active substance found to be active against food-borne bacteria *Escherichia coli* O157:H7 and *Salmonella* on the surface of freshly produced vegetables, such as lettuce, spinach and tomatoes, or raw chicken. The activity of carvacrol is also extended to drug-resistant microorganism strains with particular significance for pathogenesis as they are currently difficult to treat, such as methicillin-resistant *Staphylococcus aureus* and *S. epidermidis*. The effectiveness of carvacrol against these resistant-species was demonstrated, and MIC (Minimal Inhibition Concentration) values comprising of between 0.015 and 0.03% *v/v* for carvacrol and 0.06–0.125% for carvacrol bearing essential oils were reported, respectively. Moreover, its antimicrobial action against bacterial strains can also be shown when combined with other active substances, e.g., antibiotics [12]. Geraniol is a commercially important terpene alcohol, which occurs in the essential oils of several aromatic plants. It was found that geraniol had an antimicrobial effect on *Haemophilus influenzae*, *Streptococcus pneumoniae*, *Streptococcus pyogenes, Staphylococcus aureus Escherichia coli,* and *Salmonella* Typhimurium. An antimicrobial activity analysis showed MIC between 0.4 and 0.6 mg/L for *S. aureus* and *E. coli* [13]. Moreover, the addition of geraniol to other substances, such as antibiotics, may increase the effective action of these substances [14]. Thanks to their antibacterial properties, carvacrol and geraniol may be introduced into a coating carrier to obtain a packaging covered with an internal antibacterial coating to prolong the shelf life of food products.

The ongoing global spread of a pandemic caused by the novel coronavirus (CoV), known as SARS-CoV-2, which causes severe acute respiratory syndrome (SARS), currently poses unprecedented risks to human health and the economy. This virus belongs to a large family of enveloped viruses with +ssRNA and crown-like spikes on their spherical surfaces. CoV virion is classified as a highly pathogenic virus and has been associated with SARS-CoV-1 (2003), MERS-CoV (2012), and SARS-CoV-2 to date. The genome sequence of SARS-CoV-2 showed an 82% similarity to SARSCoV-1. The capsid confers specificity to the virus and the inner core, infectivity, and enveloped proteins associated with the virus life cycle. Enveloped viruses are diverse with reference to genome, structure, replication, pathogenicity’s, and persistence. The lipid bilayer with glycoproteins aids the virus to identify the host cell and fuse with its membrane. Peplomers assist virus attachment to the host [15]. SARS-CoV-2 transmits via human-to-human contact or contact with infected individuals or mediated through the mouth, nose, eyes, or through the inhalation of exhaled virus in respiratory droplets (coughs or sneezes from an infected person), which eventually leads to the need for ‘social distancing’ to reduce the spread of the virus. This respiratory virus has also been confirmed as being excreted in human waste (fecal-oral contamination) [15,16]. SARS-CoV-2 has infected about 19.5 million people and caused a minimum of 722,285 deaths (according to the World Health Organization [WHO] situation report accessed on 10 August 2020) [16]. To prevent the transmission of the virus, the use of synthetic polymers, such as medical masks and gloves by medical staff and health workers, and later by ordinary citizens, has become essential. For instance, demand for plastics is expected to increase by 40% in packaging and 17% in other applications, including for medical uses. Safety concerns related to shopping in supermarkets during the COVID-19 pandemic has led to a preference for fresh-food packaged in plastic containers by consumers and suppliers, as well as the use of single-use food packaging and plastic bags to carry groceries [17]. In order to address customer concerns and assure their safety, supermarkets could implement additional health safety measures, such as active packaging with antiviral properties. This safe packaging should have an internal coating to protect food products and an external coating with antiviral properties to protect customers. Additionally, this packaging material should function during storage, meaning it should offer sufficient resistance against UV aging or be shielded against ultraviolet light through an additive. This means that introducing an active substance that is resistant to UV in a coating carrier or adding a substance with shielding properties can prevent the inactivation of the coating, especially an external coating after UV-aging. It was shown that ZnO nanoparticles exhibited superior chemical stability under UV radiation, with results of a previous study confirming that nano-ZnO particles may protect their own antimicrobial property coatings [2]. Carvacrol and geraniol, from essential oils extracted from plants, were found to be effective antiviral agents that have the potential to inhibit viral spikeprotein (e.g., spikeprotein of SARS-Co-V2) [18]. This is why these substances may be used to obtain both: An internal coating with antibacterial properties and an external coating with antiviral abilities. 

Testing antiviral activity with human pathogen viruses, such as SARS-Co-V2 requires immense safety measures and are, therefore, costly. There are several common methods for the assay of antiviral activity based on in vitro or in vivo models. However, only a few laboratories are allowed to carry out tests on human viruses. Conversely, bacterial viruses, known as phages, are safe for humans. They are very specific and only attack selected hosts, do not require specialized biocontainment precautions, and are relatively easy to produce within a laboratory. Bacteriophages represent good surrogates for the study of airborne viruses and display structural features similar to many eukaryotic viruses. 

Phages are highly diversified from a genetic and morphological standpoint, thereby providing a large pool of viruses to choose from. The authors of numerous studies have analyzed tailless phages as possible model viruses for aerovirology studies: MS2 (Leviviridae family), Φ6 (Cystoviridae family), ΦX174 (Microviridae family), PM2 (Corticoviridae family), and PR772 (Tectiviridae family). It was demonstrated that these phages were similar (morphology, envelope, capsid size, and genome material) to known pathogenic viruses, such as the Human influenza A virus H1N1 (Orthomyxoviridae family) or the Newcastle disease virus (NDV; Paramyxoviridae family). It was also proved that the behavior of the influenza virus resembled that of phages PR772 and 6, while the behavior of NDV was closer to that of phages MS2 and X174. The results of many studies provide information for the selection of appropriate phage models to mimic the behavior of specific human and animal viruses in aerosols [19,20,21]. Prussin II, A.J. et al. [21] suggested that bacteriophage Phi6 was selected as a surrogate for influenza viruses and even coronaviruses. The authors designed a study to discover whether absolute humidity, relative humidity, or temperature was a better predictor of viral survival in droplets. They proved that RH was the most important factor in controlling virus infectivity. 

The first purpose of the study was to obtain an external coating based on nanoparticles of ZnO (with shielding properties), carvacrol, or geraniol that could be active against viruses, such as SARS-Co-V2. Additionally, the synergistic effect of the chosen substances in the coating was analyzed. Another aim of the study was to determine the antibacterial activity of the coatings that were created. 

## 2. Results

### 2.1. Antibacterial Analysis 

**Figure 1:**
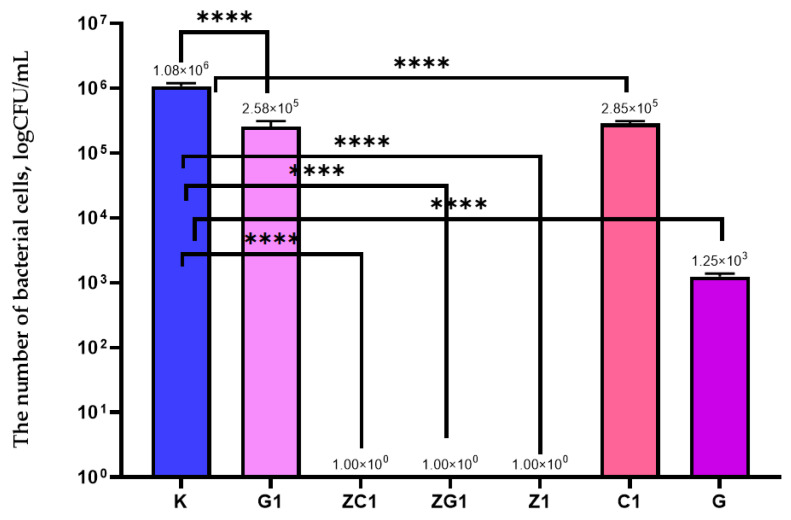
The influence of coatings on *S*. *aureus* growth. K–PE film; G1–PE film covered with the MHPC coating containing 0.0125 g of geraniol in 100 mL carrier; C1–PE film covered with the MHPC coating containing 0.0125 g of carvacrol in 100 mL carrier; G–PE film covered with the MHPC coating containing 5 g of geraniol in 100 mL carrier; Z1–PE film covered with the MHPC coating containing 0.041 g ZnO nanoparticles in 100 mL carrier; ZG1–PE film covered with the MHPC coating containing 0.041 g ZnO nanoparticles and 0.0125 g of geraniol in 100 mL carrier; ZC1–PE film covered with the MHPC coating containing 0.041g ZnO nanoparticles and 0.0125 g of carvacrol in 100 mL carrier; one-way ANOVA: ns—not significant; *—*p* < 0.05; **—*p* < 0.01; ***—*p* < 0.001; ****—*p* < −0.0001.

The result of the current study demonstrated that a decrease in the amount of ZnO nanoparticles (by 50%) in the coating (Z1) did not influence the antimicrobial properties of this coating. In the case of a methyl-hydroxy-propyl-cellulose (MHPC) coating containing 5% of geraniol (G) in the coating carrier, the number of bacterial cells decreased when compared to the control sample (K) (Figure 1). A decrease in the number of *S*. *aureus* was observed from 1.08 × 10^6^ to 1.25 × 10^3^ (CFU/mL) (Figure 1). It should also be mentioned that a characteristic aroma of geraniol of G coating was noticeable. The results of the current study confirmed that a decrease in the amount of geraniol (G1) (by 99.75%) had an influence on the activity of the coating against *S. aureus*. It was determined that the number of bacterial cells decreased from 1.08 × 10^6^ to 2.58 × 10^5^ (CFU/mL) (Figure 1). It was also important that a lowering of the scent of the coating G1 compared to G was noticed. Similar results were obtained in the case of a coating containing 0.0125 g of carvacrol in the coating carrier (C1). Statistical analysis showed that the changes in the number of bacterial cells were significant (*p* < 0.0001).

The results of this research determined that MHPC coatings obtained by the addition of nanoparticles to the carrier containing decreased (low) levels of geraniol or carvacrol (ZG1, ZC1) were found to be active against *S. aureus*. These coatings inhibited the growth of the bacterial cells. The differences between the numbers of viable cells were significant, as confirmed by a one-way ANOVA test (*p* < 0.0001).

**Figure 2:**
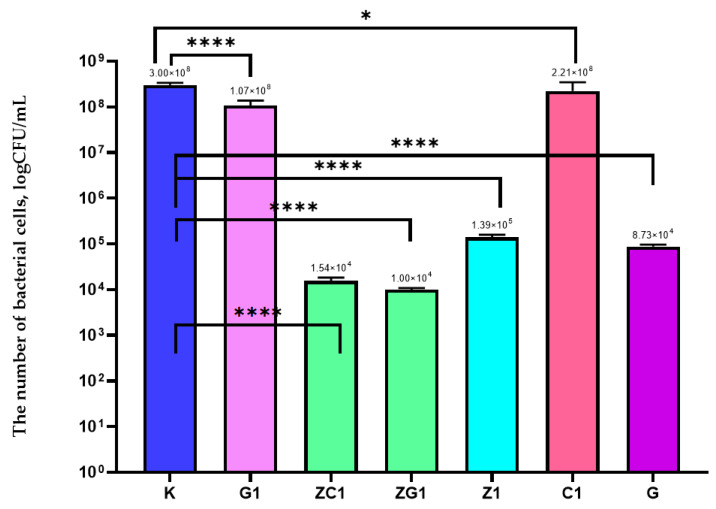
The influence of coatings on *E*. *coli* growth. K—PE film; G1–PE film covered with the MHPC coating containing 0.0125 g of geraniol in 100 mL carrier; C1–PE film covered with the MHPC coating containing 0.0125 g of carvacrol in 100 mL carrier; G–PE film covered with the MHPC coating containing 5 g of geraniol in 100 mL carrier; Z1–PE film covered with the MHPC coating containing 0.041 g ZnO nanoparticles in 100 mL carrier; ZG1–PE film covered with the MHPC coating containing 0.041 g ZnO nanoparticles and 0.0125 g of geraniol in 100 mL carrier; ZC1–PE film covered with the MHPC coating containing 0.041g ZnO nanoparticles and 0.0125 g of carvacrol in 100 mL carrier, one-way ANOVA: ns—not significant; *—*p* < 0.05; **—*p* < 0.01; ***—*p* < 0.001; ****—*p* < −0.0001.

It was demonstrated in the current study that the Z1 coating did not inhibit the growth of *E. coli* but decreased the number of bacterial cells from 3.00 × 10^8^ to 1.39 × 10^5^ (CFU/mL) (Figure 2). The current results confirmed that a decrease in the amount of ZnO nanoparticles in the coating decreased the antibacterial activity of the coating. As emphasized below (Figure 2), the antibacterial activity of G1 and C1 coatings was not noted. Alternatively, in the case of G coating, a 4 log reduction in the number of *E. coli* was observed. The results confirmed that a higher amount of geraniol in the coating caused an increase in antibacterial activity. Statistical analysis demonstrated that the differences between numbers of *E. coli* cells were significant (*p* < 0.001). Figure 2 showed that ZC1 and ZG1 coatings also decreased the number of bacterial cells. Comparing the activity of G, Z1, G1, ZG1 coatings and C1, Z1, ZC1 coatings, a synergistic effect was observed in the active substances.

**Figure 3:**
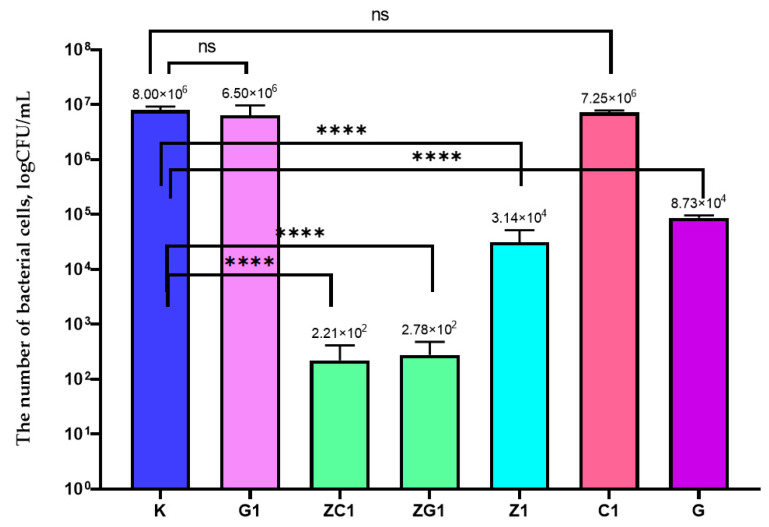
The influence of coatings on *P*. *syringae* growth. K–PE film; G1–PE film covered with the MHPC coating containing 0.0125 g of geraniol in 100 mL carrier; C1–PE film covered with the MHPC coating containing 0.0125 g of carvacrol in 100 mL carrier; G–PE film covered with the MHPC coating containing 5 g of geraniol in 100 mL carrier; Z1–PE film covered with the MHPC coating containing 0.041 g ZnO nanoparticles in 100 mL carrier; ZG1–PE film covered with the MHPC coating containing 0.041 g ZnO nanoparticles and 0.0125 g of geraniol in 100 mL carrier; ZC1–PE film covered with the MHPC coating containing 0.041g ZnO nanoparticles and 0.0125 g of carvacrol in 100 mL carrier, one-way ANOVA: ns—not significant; *—*p* < 0.05; **—*p* < 0.01; ***—*p* < 0.001; ****—*p* < −0.0001.

It was demonstrated in this study that G1 and C1 coatings did not decrease the growth of the *P*. *syringae* strain (Figure 3). A higher amount of the geraniol in the coating (G) caused an increased activity of the coating. As can be seen in Figure 3, a 3 log reduction of the number of *P. syringae* was observed for the Z1 coating. In addition, it was also observed that a reduction in the number of bacteria caused by the ZG1 coating was higher than the reduction in the number of bacterial cells after their incubation with Z1 or G1 coatings (Figure 3). As indicated by statistical analysis, a decrease in the number of bacterial cells was significant (*p* < 0.001). Similar results were obtained for the ZC1, Z1, and C1 coatings. These results confirmed the synergistic effect of the respective active substances.

### 2.2. Antiviral Analysis 

**Figure 4:**
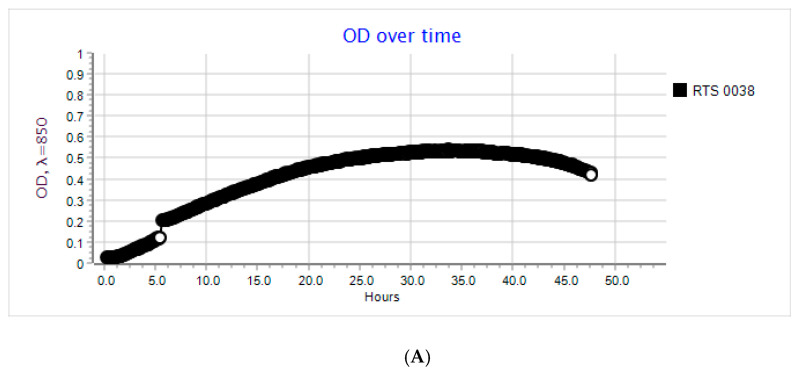

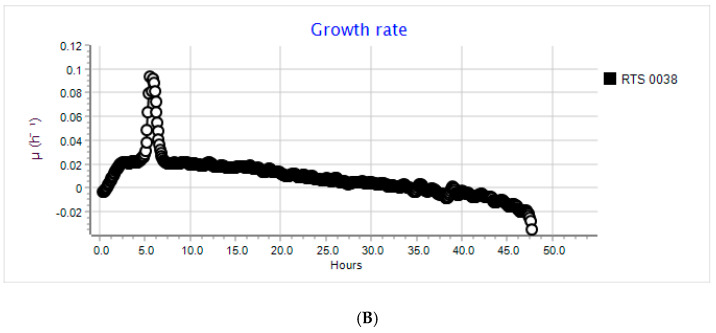
(**A**) OD over time for *P. syringe* after 48 h of incubation. Incubation devoid of phi 6 phages; OD—0.2. (**B**) The growth rate of *P. syringe* after 48 h of incubation. Incubation devoid of phi 6 phages; OD—0.2.

**Figure 5:**
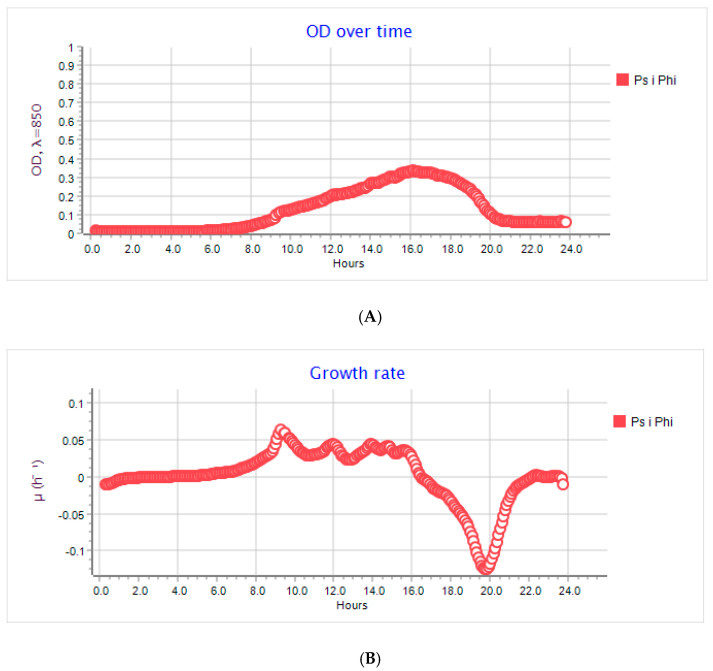
(**A**) OD over time for *P. syringe* after 48 h of incubation. Incubation with phi 6 phages, addition of the phages OD—0.2, amount of phage MOI—1; (**B**) The growth rate of *P. syringe* after 48 h of incubation. Incubation with phi 6 phages, addition of the phages OD—0.2, amount of phage MOI—1.

An analysis of the growth rate in real-time and the OD over time of the host devoid of bacteriophages and of the host that was cultivated with the active phages was performed to determine the antiviral properties of the chosen coatings. Figure 4A shows the OD over time of the *P. syringae* incubation at 28 °C. Over 48 h, a gradual increase in the number of host cells was observed. After 48 h, a OD fall was noted. As confirmed in Figure 4B, the growth rate of the bacterial cells decreased after 48 h incubation. An analysis of the OD over time and the growth rate of *P. siringae* incubated with the active phi 6 bacteriophages (Figure 5A,B) was carried out, and an OD fall was observed after 16 h of the phage incubation with the host, which showed that phages eliminated most of the bacteria. A similar result was obtained for the host culture cultivated with the phages (after their incubation with the PE film (K)). This clearly shows that the PE film was not active against phi 6 bacteriophages.

**Figure 6:**
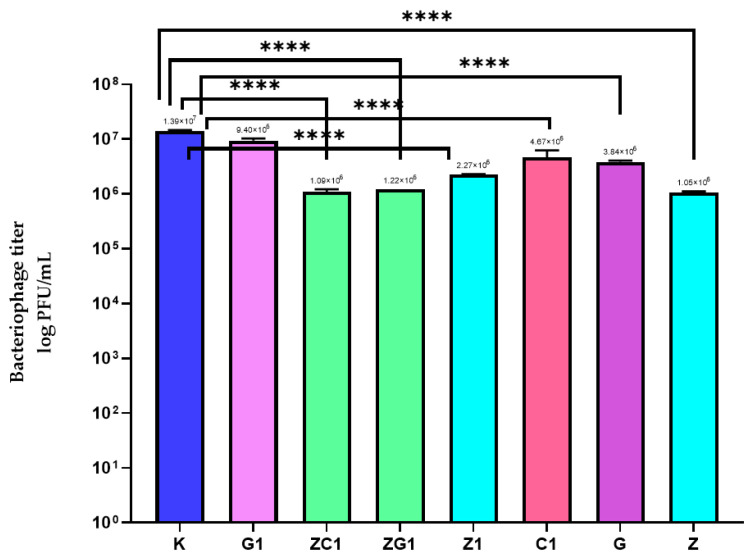
The influence of coatings on bacteriophage titer. K–PE film; G1–PE film covered with the MHPC coating containing 0.0125 g of geraniol in 100 mL carrier; C1–PE film covered with the MHPC coating containing 0.0125 g of carvacrol in 100 mL carrier; G–PE film covered with the MHPC coating containing 5 g of geraniol in 100 mL carrier; Z–PE film covered with the MHPC coating containing 0.082 g ZnO nanoparticles in 100 mL carrier; Z1–PE film covered with the MHPC coating containing 0.041 g ZnO nanoparticles in 100 mL carrier; ZG1–PE film covered with the MHPC coating containing 0.041 g ZnO nanoparticles and 0.0125 g of geraniol in 100 mL carrier; ZC1–PE film covered with the MHPC coating containing 0.041g ZnO nanoparticles and 0.0125 g of carvacrol in 100 mL carrier, one-way ANOVA: ns—not significant; *—*p* < 0.05; **—*p* < 0.01; ***—*p* < 0.001; ****—*p* < −0.0001.

**Figure 7:**
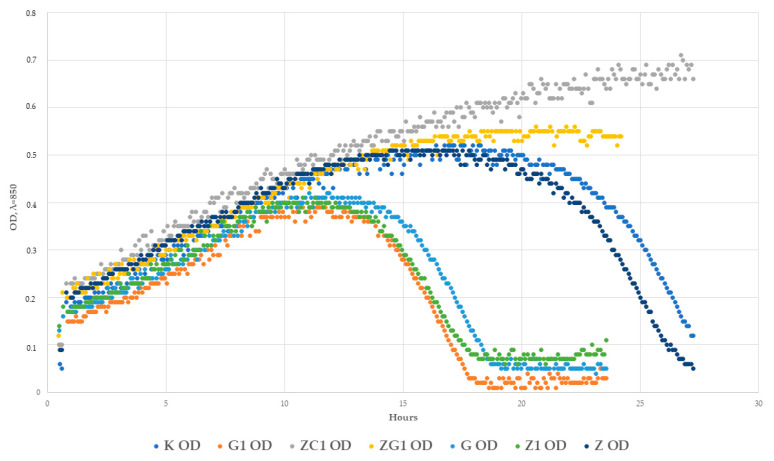
OD over time for *P. syringe* after 30 h of incubation. Incubation with phi 6 phages after their incubation with the chosen coatings; addition of phages when OD—0.2; amount of phage MOI—1.

The results of the study demonstrated that in the case of the coating containing ZnO nanoparticles (Z), a 1 log reduction in the bacteriophage titer compared to the polyethylene film (K) was observed (Figure 6). As clearly shown in Figure 7, an OD fall was observed after 16 h of phages cultivation with the host. Similar results were obtained for the host culture incubated with the active phages (Figure 5A). It was demonstrated that after 26 h cultivation, an increase in OD was observed, which meant that there were more bacteria in the culture than phages. The results proved that the incubation of the phages with the Z coating did not inactivate the phages completely but only reduced their number. It can be assumed that the Z coating demonstrated weak antiviral activity, resulting from the lack of any complete elimination of the active phage particles. 

In the case of the Z1 coating that contained the decreased amount of the ZnO nanoparticles, a smaller than 1 log reduction in the bacteriophage’s titer was noted (than when compared to the Z coating). It was shown that after 12 h of incubation of the host with phages that were incubated with the Z1 coating, these phages reduced the number of the bacteria contributing to a fall in OD (Figure 7). It was also noted that after 17 h, a re-increase in the number of bacterial cells was observed. It can be assumed that the Z1 coating also demonstrated weak antiviral activity. 

The results of the study showed that incubation of the phages with G coating or with C1 coating only marginally reduced the titer of the phages. Contradictory results were obtained in the case of the G1 coating (Figure 6), which was not active against phi 6 phages. In the case of the G coating, after a 12 h host cultivation with the phages (after their incubation with the coating), a fall in OD was observed. This clearly shows that phages reduced the amount of *P. syringae*. However, after 18 h, the OD increased, proving that the number of bacterial cells increased again. The results demonstrated that coating G showed weak activity against phi 6 bacteriaphages (Figure 7). 

An analysis of the activity of coating G1 determined that after incubation (phages with this coating G1), the bacteriophages were still active. As emphasized in Figure 7, a fall in OD was observed after 12 h incubation of the host with these phages. This conclusively showed that coating G1 was not active against the phages. 

The results of the study illustrated (Figure 6) that the incubation of the phages with the coatings ZG1 and ZC1 reduced the bacteriophage titer (1 log). Comparing the reduction of the phage titer caused by coatings ZG1 and ZC1 to the reduction caused by C1 and G1 coatings, it was observed that the addition of ZnO nanoparticles onto the coatings based on increased amounts of geraniol or carvacrol resulted in a clear increase in the antiviral activity of the coatings. An analysis of the OD of the host cultivated with the phages over time (after their incubation with the ZG1 coating) demonstrated that after 24 h, a fall in OD was noted (Figure 7). Similar results were obtained for the ZC1 coating. In this case, an OD fall was observed after 27 h of host cultivation with phages, meaning that phages reduced the number of host cells. The delayed bacteriolytic activity of phages was observed for both coatings. To summarize, it can be assumed that ZG1 and ZC1 coatings showed moderate antiviral activity, resulting from the reduction of the phage titer in the initial phase of incubation, devoid of complete elimination of any active phage particles. 

## 3. Discussion

During the pandemic, demand for plastics is expected to increase by 40% in the form of packaging [17]. Safety concerns related to shopping in supermarkets during the COVID-19 pandemic has led consumers and providers to prefer fresh-food packaged in plastic materials to avoid food contamination and to extend the shelf-life of various food products [11,17]. A packaging covered with internal active coatings may be a clear solution to this problem, but it is important that the antibacterial activity of these coatings is high.

The results of a previous study showed that MHPC coatings had no influence on the growth of *S*. *aureus* cells [2,3]. It was also determined that MHPC coatings (Z) containing 0.032 g of ZnO nanoparticles per 1 m^2^ of PE film inhibited the growth of *S*. *aureus* [2]. The results of the previous research also demonstrated that MHPC coatings had no influence on the growth of *E. coli* cells. Unlike MHPC coatings, active coating (Z) containing nanoparticles completely inhibited the growth of bacterial cells [2].

Summarizing, the coatings containing ZnO nanoparticles (Z) showed bacteriolytic activity against Gram-positive and Gram-negative bacterial cells [2,3]. The boxes containing PE films covered with Z coating were found to be the best packaging material to extend the quality and freshness of cod fillets after 144 h storage at 5 °C [11]. As a result of the study, a decrease in the number of nanoparticles in the coating caused a decrease in the activity of the coating against Gram-negative bacteria, but not against Gram-positive bacteria. The results showed here are comparable with those reported by Esmailzadeh H. et al. [22] and Gandhi R.R. et al. [23], who proved that Gram-positive bacteria were more sensitive to ZnO nanoparticles than to Gram-negative bacteria. Results that found contrary results were obtained by Sharma D. et al. [24], whose coatings exhibited greater antibacterial activity against *E. coli* bacterium than against *S. aureus*. The coatings containing geraniol (G) showed bacteriostatic activity against Gram-positive and Gram-negative bacteria, though these had a noticeable geraniol aroma. As reported by the authors [13,14,15], the aroma of geraniol was noticeable because both this substance and carvacrol are volatile molecules, which evaporate easily, and their vapor phase has shown antimicrobial activity. A reduction in the amount of geraniol in the active coating (G1) caused a lessening of the coating aroma. Similarly, the addition of a very small amount of carvacrol to the coating carrier (C1) also limited the scent of the coating. Unfortunately, coatings G1 and C1 (containing a very small number of active substances) were found to not be active against Gram-positive and Gram-negative bacteria. This was caused by not having enough active substances in the coatings. In the case of Gram-negative bacteria, the addition of an increased amount of ZnO nanoparticles to the coatings containing geraniol or carvacrol (but a very small amount of these substances that caused a lack of the antibacterial coating activity) had an influence on an increase in the coating’s activity. Coatings ZC1 and ZG1, which contained an increased amount of geraniol and carvacrol, were found to be active, proving that there is a synergistic effect between these active substances. In the case of Gram-positive bacteria, coatings ZG1 and ZC1 showed bacteriolytic activity that could have been caused by ZnO nanoparticles. To summarize, PE films covered with an internal MHPC coating with a very small amount of ZnO nanoparticles and geraniol or carvacrol were found to be the best packaging material to extend the quality and freshness of food products. The same coatings (ZG1 and ZC1) may be used as external coatings to obtain single-use food packaging and plastic bags to carry groceries. These coatings would be active thanks to the shielding properties of the ZnO nanoparticles. The author’s previous study highlighted the UV- blocking properties of nanoZnO [2,11]. The coatings G, Z, and Z1 offered weak activity, but coatings ZC1 and ZG1 showed moderate activity against enveloped by a lipid phi 6 phage layer. It can be surmised that coatings ZG1 and ZC1, which are active against phi 6 bacteriophage, would also be active against SARS-CoV-2, transmitted via respiratory droplets. Many authors have indicated that bacteriophages can be used as a model surrogate for eucaryotic viruses [19]. Pan et al. [19] used bacteriophages to develop a model to assess how infectious viruses might distribute in airborne particles. Prussin II, A.J [21] suggested that the Phi6 bacteriophage was selected as a surrogate for influenza viruses and even coronaviruses. The authors designed a study to determine whether absolute humidity, relative humidity, or temperature was a better predictor of virus survival in droplets. The packaging materials covered with the ZG1 and ZC1 coatings (as external coatings) could protect the consumers and limit the spread of SARS-CoV-2. The SARS-Co-V2 spread is an immense risk to human health, and in the future, there will also be more epidemic or pandemic outbreaks due to the ever-changing anthropogenic activities affecting the ecological balance. In this context, the possibility of viral transmission through packaging materials is a very important point of concern, and their inactivation through the use of active packaging will play a major role in the curbing of their spread into to general population.

## 4. Materials and Methods

### 4.1. Materials

The bacterial strains and phage phi6 used in this experiment were obtained from a collection from the Leibniz Institute DSMZ (Deutsche Sammlung von Mikroorganismen und Zellkulturen, Braunschwelg, Germany). The organisms used in this study were *S*. *aureus* strain DSMZ 346, *E*. *coli* DSMZ 498 and *P*. *syringae* van Hall 1902 DSM 21482. As a SARS-CoV2 surrogate, phage phi 6 DSM-21518 was used.

Polyethylene films (A4, 50 μm) (KB FOLIE) were obtained from a KB FOLIE (Warsaw, Poland). The MHPC (Chempur, Piekary Śląskie, Poland) was used as a coating carrier, as well as Zinc Oxide AA 44899, (~70 nm), geraniol (Sigma-Aldrich, Poznań, Poland), and carvacrol (Avitale Pure Liquids, Białystok, Poland), which were used as an antibacterial and antiviral substance. TSB, TSA, and Luria-Bertani (LB) broth (Merck, Darmstadt, Germany) were used to determine the antimicrobial properties of any coatings. All mediums were prepared according to the Merck protocol (all mediums were weighed according to the manufacturer’s instructions, suspended in 1000 mL of distilled water, and autoclaved at 121 °C for 15 min).

### 4.2. Coating Preparation

(1) 4 g of MHPC was introduced into 100 mL of water. The mixture was mixed for 1 h using a magnetic stirrer (Ika, Warsaw, Poland) at 1500 rpm. Then, 95 g of MHPC was mixed with 5 g of geraniol and homogenized (1000 rpm) (Heidolph, Sigma-Aldrich, Poznań, Poland). A mixture of 5% carvacrol in MHPC was not obtained (G).
(2) 8 g of MHPC was introduced into 200 mL of water. The mixture was mixed for 1 h using a magnetic stirrer (Ika, Warsaw, Poland) at 1500 rpm. Then 99.9875 g of MHPC was mixed with 0.0125 g of geraniol (G1), and 99.9875 g of MHPC was mixed with 0.0125 g of carvacrol (C1) (separately) and homogenized (1000 rpm) (Heidolph, Sigma-Aldrich, Poznań, Poland).
(3) 0.082 g of ZnO nanoparticles were introduced into 50 mL of water. As a first step, the mixture was mixed for 1 h using a magnetic stirrer (450 rpm). Next, the mixture was sonicated for 30 min. (sonication parameters: Cycle: 0.5; amplitude: 20%), while at the same time, a 2nd mixture (4 g of MHPC into 50 mL) was prepared as described above. A ZnO nanoparticle solution was introduced into the MHPC mixture (Z) and sonicated for 10 min.
(4) 0.041 g of ZnO nanoparticles were introduced into 50 mL of water. Then, the mixture was mixed for 1 h using a magnetic stirrer (450 rpm). Next, the mixture was sonicated for 30 min (sonication parameters: Cycle: 0.5; amplitude: 20%), while at the same time, the 2nd mixture (4 g of MHPC into 50 mL) was prepared as described above. The ZnO nanoparticle solution was introduced into the MHPC mixture (Z1) and sonicated for 10 min (sonication parameters as described above).
(5) 0.082 g of ZnO nanoparticles were introduced into 100 mL of water. Initially, the mixture was mixed for 1 h using a magnetic stirrer (450 rpm), the mixture was then sonicated for 30 min (sonication parameters: Cycle: 0.5; amplitude: 20%), while at the same time, the 2nd and the 3rd mixtures (99.9875 g of MHPC was mixed with 0.0125 g of geraniol and 99.9875 g of MHPC was mixed with 0.0125 g of carvacrol, separately) were prepared as described above. Then 50 mL of water solution of the nanoparticles was introduced into 50 mL of the 2nd mixture (Z1G1), and 50 mL of nano ZnO solution was introduced into 50 mL of the 3rd mixture (Z1C1). The mixtures were sonicated (sonication parameters: Cycle: 0.5; amplitude: 20%; time: 10 min).

Polyethylene (PE) films were coated using Unicoater 409 (Erichsen, Hemer, Germany) at a temperature of 25 °C with a 40 μm diameter roller. The coatings were dried for 10 min at a temperature of 50 °C. 1.6 g layers of methyl-hydroxy-propyl-cellulose per 1 m^2^ of PE were obtained [3]. PE films that were not coated were control samples (K).

**Figure 8:**
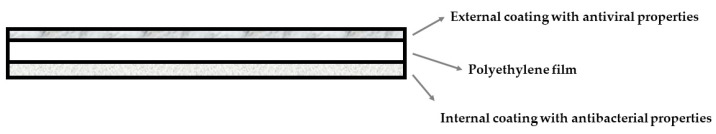
Multilayer active polyethylene packaging.

PE films were covered with the coatings on one side of PE, but the active substances may be used to obtain both: An internal coating with antibacterial properties and an external coating with antiviral abilities (Figure 8).

The film samples were then cut into square shapes (3 cm × 3 cm).

### 4.3. Antibacterial Analysis

In the case of *S. aureus* and *E. coli* strains, the antibacterial properties of the covered films compared to the non-covered were carried out according to the ISO 22196-2011 standard [25]. In the case of the *P. syringae* strain, the antimicrobial properties of non-covered and covered films were carried out according to the ASTM E 2180-01 standard [26].

### 4.4. Antiviral Analysis

As a 1st step of the experiments, the bacteriophage phi6 was purified according to Bhetwal et al. [27]. Then the phage lysate was prepared according to Bonilla et al. [28]. The antiviral properties of the covered films were compared to the non-covered films and were carried out according to a modified ISO 22196-2011 standard [25]. Finally, phage amplification was carried out using the Skaradzińska et al. method [29].

To analyze the *P. syringae* growth rate in real-time, after its incubation with the active coatings, phi6 lysate was incubated with the coatings (each active coating separately) according to the ISO 22196-2011 standard, while phi6 lysate was incubated with a PE film devoid of coating at the same time. The LB broth was introduced into 2 BioSan bioreactors (BS-010160-A04, BioSan, Riga, Latvia). Then, bacterial overnight culture was added to 30 mL of LB broth and incubated at 28° until OD = 0.2 (optical density). Two phi 6 bacteriophage lysates were amplified in respective host bacteria (1 lysate—after incubation with the PE film, 1 lysate—after incubation with the chosen coating). Next, 11 µL of phage lysate (MOI = 1, 1 phage per 1 bacterial cell) were added to a host culture (OD = 0.2) and incubated for 24 h at 28°. Two tests were carried out simultaneously, and it was possible to analyze 1 active coating during these experiments, with the activity of the remaining coatings also analyzed.

### 4.5. Statistical Analysis

Statistical significance was noted using an analysis of variance (One-way ANOVA). The values were considered significantly different when *p* < 0.05. All analyses were performed with GraphPad Prism 8 (GraphPad Software, San Diego, CA, USA).

## 5. Conclusions

The coatings containing ZnO nanoparticles (Z) showed bacteriolytic activity against Gram-positive and Gram-negative bacterial cells. As a result of the study, a decrease in the number of nanoparticles in the coating caused a decrease in the activity of the coating against Gram-negative bacteria, though not against Gram-positive bacteria. The coatings containing geraniol (G) showed bacteriostatic activity against Gram-positive and Gram-negative bacteria, though a characteristic aroma of geraniol was noticeable. A reduction in the amount of geraniol in the active coating (G1) caused a lowering of the scent of the coating. Unfortunately, coatings G1 and C1 (containing a very small number of active substances) were found to not be active against Gram-positive and Gram-negative bacteria. The ZC1 and ZG1 coatings containing an increased amount of geraniol and carvacrol and nanoZnO were found to be active, proving that there is a synergistic effect between these active substances. In the case of Gram-positive bacteria, coatings ZG1 and ZC1 showed bacteriolytic activity. To summarize, PE films covered with an internal, MHPC coating with a very small amount of ZnO nanoparticles, as well as geraniol or carvacrol, may be used as an effective packaging material to extend the quality and freshness of food products. The same coatings (ZG1 and ZC1) could also be used as external coatings with antiviral properties. ZC1 and ZG1 coatings showed moderate activity against phi 6 phage, selected as a surrogate for viruses, such as coronaviruses. It may be assumed that coatings ZG1 and ZC1 will also be active against SARS-CoV-2 transmitted via respiratory droplets. There is the possibility that SARS-CoV-2 can also be transmitted through packaging materials, and active packaging materials may offer the opportunity to limit its transmission.

## Data Availability

Not applicable.
